# Surviving the Scene in Civilian Penetrating Brain Injury: Injury Type, Cause and Outcome in a Consecutive Patient Series in Austria

**DOI:** 10.3389/fsurg.2022.923949

**Published:** 2022-06-28

**Authors:** Franz Marhold, Florian Scheichel, Barbara Ladisich, Philip Pruckner, Elisabeth Strasser, Melanie Themesl, Karl Ungersboeck, Branko Popadic

**Affiliations:** ^1^ Karl Landsteiner University of Health Sciences, Krems, Austria; ^2^ Department of Neurosurgery, University Hospital St. Poelten, St. Poelten, Austria

**Keywords:** penetrating brain injury, low velocity, high velocity, traumatic brain injury, firearm injury

## Abstract

**Background:**

Penetrating brain injury (PBI) is a heterogeneous condition with many variables. Few data exist on civilian PBI. In some publications, PBI differentiation between low-velocity injury (LVI) and high-velocity injury (HVI) is made, but exact definitions are not given yet. The incidence of PBI depends heavily on the country of origin. Furthermore, captive bolt pistol (CBP) injuries represent a rare type of LVI and almost no reports exist in the human medical literature. Treatment of PBI has been controversially discussed due to high morbidity and mortality with results varying considerably between series. Prognostic factors are of utmost importance to identify patients who presumably benefit from treatment.

**Methods:**

A retrospective, single-center analysis of a consecutive patient series was performed from September 2005 to May 2018. We included all patients with PBI who reached our hospital alive and received any neurosurgical operative procedure.

**Results:**

Of 24 patients, 38% died, 17% had an unfavourable outcome, and 46% had a favourable outcome. In total, 58% of patients with PBI were self-inflicted. Leading causes of injury were firearms, while captive bolt pistols were responsible for 21% of injuries. LVI represented 54%, and HVI represented 46%. The outcome in HVI was significantly worse than that in LVI. A favourable outcome was achieved in 69% of LVI and 18% of HVI. Low GCS and pathological pupillary status at admission correlated significantly with an unfavourable outcome and death.

**Conclusions:**

PBI is a heterogeneous injury with many variables and major geographical and etiological differences. Differentiation between LVI and HVI is crucial for decision-making and predicting outcomes. In patients presenting with object trajectories crossing the midline, no favourable outcome could be achieved. Nevertheless, in total, a favourable outcome was possible in almost half of the patients who succeeded in surgery.

## Introduction

Penetrating brain injury (PBI) in western European countries in civilian settings is rare and few reports exist (1–3). Furthermore, it is a heterogeneous condition with different variables predicting the clinical course. Injury mechanism, location, the extent of injury, aetiology, and different causes have all been taken into account for decision-making in a mostly urgent setting. In general, PBI is thought to be associated with high morbidity and mortality.

In craniocerebral gunshot wounds, up to 76% of patients die at the scene or during transport and less than 20% of patients receive any in-hospital neurosurgical treatment (4–8). Although morbidity and mortality are high, a favourable outcome is possible in about 19% of these patients (9).

While it is differentiated between low-velocity injury (LVI) and high-velocity injury (HVI) in PBI in some papers, exact definitions of LVI and HVI remain unclear and are mostly arbitrary (10, 11). The cut-off points for the velocity of the penetrating object vary from 120 to 914 m/s (11, 12). This has led to considerable differences in their definitions of LVI and HVI between reports. Considering the complex physical mechanisms in PBI, a more general approach has been proposed, defining LVI and HVI in PBI by the nature of the injury. LVI causes highly localized tissue damage along the object's trajectory, while HVI is characterized by penetration generating both permanent and temporary cavities, resulting in damage beyond the immediate contact region between the projectile and tissue (12, 13). Consequently, the outcome in LVI is generally seen as more favourable than in HVI (14, 15).

HVI is mostly caused by firearms, and therefore, its incidence depends heavily on the country and its laws regarding gun control. In the United States, the calculated rate of total gun deaths per 100,000 people amounted to 11.28 in 2015 (16). In the same year in Austria, the rate of total gun deaths per 100,000 people was 2.69 (17). Since the enactment of more stringent firearm legislation in 1997 by the European Union, this rate has decreased significantly between the beginning and the end of the nineties (18). Moreover, it still decreases within the last decade (17).

LVI is typically a result of accidents, hence occurring more often in countries with low standards in work and traffic safety regulations (14, 15). Captive bolt pistol (CBP) injuries represent a rare type of PBI. This device is used in animal slaughter, where a bolt is propelled through the skull into the brain with an average velocity of about –75 m/s and is then retracted by a spring (19). In contrast to popular belief, the purpose of most CBP is not to kill the animal but to stun it prior to slaughter by inducing unconsciousness. Only a few reports exist in the literature about its misuse in a suicidal or homicidal manner, which mostly comes from European countries where this slaughter technique is frequently used (19). Considering the nature of this injury, CBP injuries should be categorized as LVI.

Treatment of PBI has been controversially discussed due to high morbidity and mortality with results varying considerably between series. Prognostic factors are of utmost importance to identify patients who presumably benefit from treatment, especially if they present themselves in a bad clinical condition.

Thus, we add a single-centre experience on civilian PBI of a European country to the sparse reports in the literature, highlighting the heterogeneity of this injury pattern and including detailed differentiation between LVI and HVI.

## Methods

A retrospective analysis of a consecutive patient series with PBI was performed from September 2005 to May 2018. We included all patients who survived the scene, reached our hospital alive, and received any neurosurgical operative procedure. All patients were treated at the Department of Neurosurgery of the University Hospital St. Poelten (Karl Landsteiner University of Health Sciences), Austria, which serves as a level-1 trauma center. The study was conducted according to Austrian law and in accordance with the Declaration of Helsinki for good clinical practice.

Medical records were analysed, and variables such as demographics, incident-related data, clinical data [Glasgow Coma Scale (GCS), pupillary status at arrival, and type of surgery], and radiological findings [trajectory of penetration, ventricles and cisterns at admission, cranial computed tomography (CCT), infections, and Glasgow Outcome Score (GOS)] at follow-up were evaluated. The trajectory of penetration could either be unilateral (ipsilateral) or bilateral (bihemispheric), which means that the lesion crossed the midline. CT angiography was not routinely performed, and analysis was omitted.

The mean follow-up was 58 months with a range of 1–112 months. The favourable outcome was designated as GOS 4–5, the unfavourable outcome was designated as GOS 2–3, and death was designated as GOS 1.

The injury type of PBI was divided into two categories, low-velocity injury (LVI) and high-velocity injury (HVI), as previously reported (12, 13). The velocity of the penetrating object in high-velocity injuries was at least above 120 m/s. Firearm injuries were categorized as high-velocity injuries, and all other penetrating injuries such as impalements, nailgun, and captive bolt pistols were categorized as low-velocity injuries. In captive bolt pistols, the velocity of the penetrating object (firing pin) ranged between 55 and 70 m/s.

Statistical analysis was performed using commercially available software (SPSS, IBM, Armonk, NY, Version 21). For categorical variables, the chi-square test was used. Kruskal–Wallis test and Mann–Whitney *U* test were used for analysing continuous ordinal variables. A *p*-value of <0.05 was considered statistically significant.

## Results

In this study, a total of 24 patients were included. Patient characteristics, clinicoradiological parameters, outcomes, and detailed differentiation between LVI and HVI are presented in Tables 1–3.

**Table 1:** Patient characteristics.

	*n* or mean	%
Patients	24	100
Male	23	96
Female	1	4
Age		
Mean in years (range)	52 (8–78)	
Follow-up		
Mean in months (range)	58 (1–112)	
Aetiology		
Self-inflicted	14	58
Accident	8	33
Assault	2	8
Cause		
Firearm	11	46
Impalement	6	25
Captive bolt pistol	5	21
Nailgun	2	8
Injury type		
Low velocity	13	54
High velocity	11	46
Midline shift		
Mean in mm (range)	4 (0–12)	
Surgical intervention		
Craniotomy and debridement	9	38
Frontobasal repair	6	25
Decompressive craniectomy	5	21
Wound debridement	4	17
Infection	4	17

**Table 2:** Clinicoradiological parameters in relation to injury type.

	Low velocity	High velocity	*p*-Value
Patients	13 (54%)	11 (46%)	
Aetiology			**0** **.** **025**
Accident	7 (54%)	1 (9%)	
Self-inflicted	6 (46%)	8 (73%)	
Assault	0 (0%)	2 (18%)	
GCS			**0** **.** **006**
13–15	9 (69%)	1 (9%)	
9–12	0 (0%)	1 (9%)	
3–8	4 (31%)	9 (82%)	
Pupillary status			nsf
Pathological	2 (18%)	4 (44%)	
Non-pathological	9 (82%)	5 (56%)	
Trajectory			**0** **.** **008**
Unilateral	12 (92%)	4 (36%)	
Bilateral	1 (8%)	7 (64%)	
Cisterns			Nsf
Present	11 (85%)	6 (64%)	
Compressed	2 (15%)	4 (36%)	
GOS			**0** **.** **003**
Favourable	9 (69%)	2 (18%)	
Unfavourable	3 (23%)	1 (9%)	
Death	1 (8%)	8 (73%)	

*Bold values indicate significant findings.*

**Table 3:** Clinicoradiological parameters in relation to outcome (GOS).

	Favourable	Unfavourable	Death	*p*-Value
Patients	11 (46%)	4 (17%)	9 (38%)	
Aetiology				nsf
Accident	6 (75%)	0 (0%)	2 (25%)	
Self-inflicted	4 (29%)	4 (29%)	6 (43%)	
Assault	1 (50%)	0 (0%)	1 (50%)	
Cause				**0** **.** **009**
Nailgun	2 (100%)	0 (0%)	0 (0%)	
Impalement	5 (83%)	0 (0%)	1 (17%)	
Firearm	2 (18%)	1 (9%)	8 (73%)	
Captive bolt pistol	2 (40%)	3 (60%)	0 (0%)	
Injury type				**0** **.** **003**
High velocity	2 (18%)	1 (9%)	8 (73%)	
Low velocity	9 (69%)	3 (23%)	1 (8%)	
Initial GCS				**0** **.** **008**
13–15	8 (80%)	0 (0%)	2 (20%)	
9–12	1 (100%)	0 (0%)	0 (0%)	
3–8	2 (15%)	4 (31%)	7 (54%)	
Pupillary status				**0** **.** **025**
Pathological	1 (17%)	0 (0.0%)	5 (83%)	
Non-pathological	9 (64%)	2 (14%)	3 (21%)	
Trajectory				**<0.001**
Unilateral	11 (69%)	3 (18%)	2 (13%)	
Bilateral	0 (0%)	1 (13%)	7 (88%)	
Ventricles				nsf
Normal	6 (67%)	1 (11%)	2 (22%)	
Pathological	5 (33%)	3 (20%)	7 (47%)	
Cisterns				nsf
Present	9 (50%)	3 (16.7%)	6 (33.3%)	
Compressed	2 (33.3%)	1 (16.7%)	3 (50%)	

*Bold values indicate significant findings.*

### Patient Characteristics

**Figure 1:**
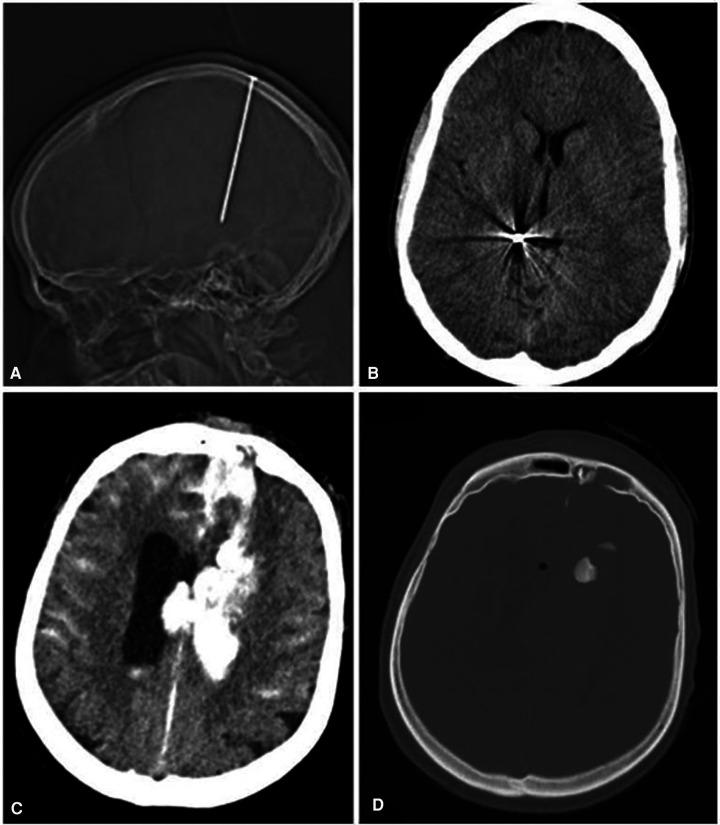
(**A**) Low-velocity injury (LVI). (**B**) Accidental nailgun injury; note that there are no signs of parenchymal damage surrounding the nail. (**C,D**) Captive bolt pistol injury. Note the pathognomic finding with a wide hemorrhagic wound canal and corresponding bone fragment at the end of the canal with the absence of metallic fragments or exit sites (19–21).

**Figure 2:**
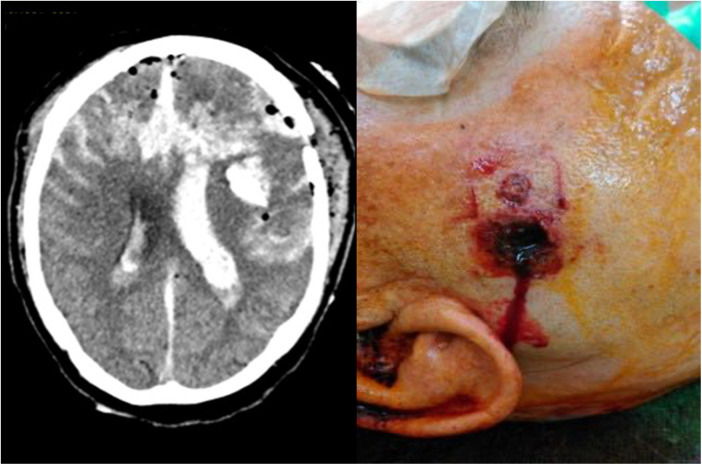
High-velocity injury (HVI); self-inflicted gunshot wound; (left) bilateral trajectory on CCT, (right) muzzle stamp.

Details of patient characteristics are given in Table 1. One female patient (4%) and 23 male patients (96%) were included. The median age was 52 years with a range from 8 to 78 years. Aetiology of injury was a self-inflicted injury in 14 cases (58%), accidents in 8 cases (33%), and assaults in 2 cases (8%). Leading causes of injury were firearms in 11 cases (46%), followed by impalement in 6 cases (25%), captive bolt pistols in 5 cases (21%), and nailguns in 2 cases (8%). Impaling objects were two iron bars, one lily stem, one piece of wood, one propeller, and one drilling machine. According to the nature of the injury, patients were divided into LVI (*n* = 13; 54%) and HVI (*n* = 11; 46%). Penetrating injuries through impalement, nailguns (Figures 1A,B), and captive bolt pistols (Figures 1C,D) were classified as LVI, while penetrating injuries by firearms (Figure 2) represented HVI. Surgical interventions were craniotomies with debridement, frontobasal repair, decompressive craniectomy, and wound debridement. All surgical interventions were performed by a neurosurgical resident under the strict supervision of a consultant, except for frontobasal repair that was carried out by a senior neurosurgical consultant. As each patient was treated at a level-1 trauma centre and received an adequate standard of care depending on the injury type, the difference between these interventions was not analysed further. Infections occurred in four cases (18%), of which one case was a nailgun injury, two cases were impalement injuries, and one case was a CBP injury. Seizures occurred in 6 patients out of 15 survivors (40%) with 1 early (two weeks postoperatively) and 5 late seizure onsets (1–4 years after PBI). In all cases, seizures could be controlled with a single antiepileptic medication.

### Detailed Analysis of Low-Velocity and High-Velocity Injuries According to Clinicoradiological Parameters

Clinicoradiological parameters and outcomes in LVI and HVI were analysed and are depicted in Table 2. LVI and HVI showed significant differences regarding the aetiology of the injury (*p* = 0.025). LVI was seen in accidents in seven cases (54%) and in self-inflicted injuries in six cases (46%). Assaults were never associated with LVI. HVI was seen in accidents in one case (9%), in self-inflicted injuries in eight cases (73%), and in assaults in two cases (18%).

Furthermore, HVI was significantly associated with low GCS scores at admission (*p* = 0.006). In LVI, nine patients (69%) had a GCS of 13–15 and four patients (31%) had a GCS of 3–8. In HVI, one patient (9%) presented with a GCS of 13–15, one patient (9%) presented with a GCS of 12–9, and 9 patients (82%) presented with a GCS of 3–8.

The radiological trajectory of the penetration was significantly more often unilateral (ipsilateral) than bilateral (lesions crossing the midline) in LVI (*p* = 0.008). A bilateral trajectory in LVI could only be seen in one patient (8%). In HVI, a unilateral trajectory was seen in four patients (36%) and a bilateral trajectory was seen in seven patients (64%).

The outcome in HVI was significantly worse than that in LVI (*p* = 0.003). In LVI, a favourable outcome was achieved in nine patients (69%), an unfavourable outcome was achieved in three patients (23%), and one patient (8%) died. In HVI, a favourable outcome was achieved in two patients (18%), an unfavourable outcome was achieved in one patient (9%), and eight patients (73%) died.

No statistical significance was found regarding pupillary status on admission, ventricle size, or appearance of basal cisterns on CCT between LVI and HVI.

### Glasgow Outcome Score According to Clinicoradiological Parameters

Clinicoradiological parameters were analysed in relation to the outcome (Table 3), and significant differences were found. A favourable outcome was achieved in 11 patients (46%), an unfavourable outcome was achieved in 4 patients (17%), and 9 patients (38%) died. The cause of injury showed significant statistical differences (*p* = 0.009). Both patients with nailgun injuries (100%) showed a favourable outcome. If impalement was the cause, five patients (83%) showed a favourable outcome and one patient (17%) died. When firearm weapons were used, two patients (18%) showed a favourable outcome, one patient (9%) showed an unfavourable outcome, and eight patients (73%) died. If captive bolt pistols were used, two patients (40%) had a favourable outcome and three patients (60%) survived with an unfavourable outcome.

In patients with HVI, two (18%) had a favourable outcome, one (9%) had an unfavourable outcome, and eight patients (73%) died. In patients with LVI, nine (69%) had a favourable outcome, three (23%) had an unfavourable outcome, and one patient (8%) died.

High GCS at admission was significantly associated with a favourable outcome and low GCS with an unfavourable outcome or death (*p* = 0.008). In mild TBI, eight patients (80%) had a favourable outcome and two patients (20%) died. In moderate TBI, the only patient in our series achieved a favourable outcome. In severe TBI, two patients (15%) achieved a favourable outcome, while four patients (31%) had an unfavourable outcome and seven patients (54%) died.

Pupillary status at admission was correlated significantly with the outcome (*p* = 0.025). If pathological, one patient (17%) had a favourable outcome and five patients (83%) died. If no pathological pupils were present, nine patients (64%) were found to have a favourable outcome, two patients (14%) had an unfavourable outcome, and three patients (21%) died.

The radiological penetrating trajectory within the brain on CCT correlated significantly with death (*p* < 0.001). Patients with unilateral trajectory had a favourable outcome in 11 cases (69%), unfavourable outcome in three cases (18%), and died in two cases (13%). If a bilateral trajectory was present, no favourable outcome could be achieved, one patient (13%) had an unfavourable outcome, and seven patients (88%) died. Furthermore, all infections occurred in patients with a favourable outcome. It is important to note that all of these patients suffered from LVI.

No statistical differences could be detected regarding the aetiology of the incident, the size of the ventricles, or the appearance of basal cisterns.

## Discussion

To the best of our knowledge, only few papers have investigated the clinical course of PBI in a civilian European population (1–3). Up to 76% of patients with PBI die at the scene, while less than 20% of patients receive any neurosurgical treatment (4–8). After surviving the scene and the transport, morbidity and mortality are high. However, a favourable outcome is possible in some patients (2, 7, 9, 22, 23). Attending physicians face the difficult task of identifying those patients. We therefore conducted this retrospective analysis of patients who reached our hospital alive and received any surgical treatment in a solely civilian setting.

We included 24 patients, of whom 38% (9/24) died, 17% (4/24) had an unfavourable outcome, and 46% (11/24) had a favourable outcome. Almost half of the patients with PBI and a favourable outcome is an interesting finding and reflects the different nature of PBI in a civilian setting compared to combat-related PBI, where the main causes of PBI are firearm or blast injuries with subsequent worse outcomes.

Incidence, aetiology, and outcome of PBI seem to differ widely between studies depending on its country of origin. A significant increase of civilian firearm injuries in general but also of penetrating firearm injuries to the head and neck within the last two decades was recently reported from a Scandinavian trauma center and is of note (24). Pena-Martínez reported an alarmingly 800% increase of musculoskeletal gunshot wounds over a 4-year period in one of the most violent cities of the world in Central America (10). However, the incidence of civilian firearm injuries to the head in western Europe is fortunately still rare (3), but it is of importance to be engaged with civilian PBI data in contrast to combat-related PBI (2), as many variables such as patient cohorts, trauma circumstances, injury type, cause, and subsequently the outcome are different and distinguishable.

### Patient Characteristics

Gender distribution in our study was comparable to other studies, with a predominantly male study population (9, 23, 25, 26). Aetiology showed that most PBIs in our study were self-inflicted or a result of accidents. As expected, the leading cause of PBI in our study was firearm injures with an incidence of 46%. In contrast, Muehlschlegel reported in a study from the United States that 94% of PBIs were a result of firearm injuries (23). This highlights major geographical differences. Another cause for PBI in our series was the use of CBP. Interestingly, CBP was used in 21% of our patient cohort, while in general CBP is seen as a very rare type of PBI. Only a few reports on CBP injuries exist, which exclusively come from European countries, where this animal slaughter technique is widely used and licence policy for these tools is generally less strict (19–21). In our series, CBP injuries were exclusively used in self-inflicted injuries and showed typical findings on CCT with a displaced skull fragment into the parenchyma (Figures 1C,D). A mortality of 60%–90% has been reported in the literature (19, 20). This is in strong contrast to our series with no mortality during the follow-up and a favourable outcome in 40% of patients.

### LVI and HVI According to Clinicoradiological Parameters

To the best of our knowledge, LVI and HVI have rarely been compared in the literature. Our series showed statistical significance in aetiology, GCS at admission, penetrating trajectory, and outcome. The available literature on LVI consists mostly of case reviews, while larger studies are still lacking. However, most of our findings seem to coincide with these reports (15, 27).

Accidents were the most common reason for LVI (*n* = 7, 54%), with all cases being a result of impalements and nailgun injuries. This corresponds to reports from the literature, where LVI is generally seen as a result of accidents and occurs more frequently in countries with low standards in work and traffic safety regulation (14, 15). In HVI, self-inflicted injuries (*n* = 8, 73%) were the most common aetiology in our series. Another study from Austria showed similar results, with 83% of the penetrating gunshot wounds to the head being self-inflicted (28). In contrast, Deng reported in a nationwide study from the United States on firearm-related PBI that 42% were self-inflicted and 49% were a result of an assault (26), which, once again, demonstrates geographical differences in PBI.

Furthermore, HVI was significantly more often associated with severe TBI. The bilateral penetrating trajectory was also significantly more common in HVI, reflecting the nature of firearm injuries with subsequent worse outcomes. CT angiography in the urgent setting is of great importance to assess possible vascular injury and a low threshold for angiography at diagnosis is proposed (29). Also, in the long term, CT angiography is useful in detecting vascular injuries such as pseudoaneurysms, carotid-cavernous fistulas, or dural arteriovenous fistulas.

Interestingly, differences in pupillary status, ventricle size, and presentation of basal cisterns were not found to be significant. This could be a bias due to the limited number of included patients, as one would assume differences between the two groups.

When comparing infection rates between injury types, it became evident that all four cases (17%) occurred in patients with LVI. One possible explanation could be the greater risk of retention of contaminated material in LVI as described in a review by Bayston et al. (30). Another explanation could be that due to the high mortality in HVI, only patients with LVI developed an infection in our study collective. In PBI, all our patients received immediately antibiotics at admission. Nevertheless, these patients were able to achieve favourable outcomes. This underlines the importance of adequate prophylactic and therapeutic antibiotic use as well as meticulous surgical removal of contaminated material when treating patients with PBI.

Another important issue to deal with is seizures. In total, 40% (6/15) of patients in our series developed seizures during their course. The onset is in line with the published data, although very few reports exist (29, 31–33). Shaw reported seizure onset below 10% within the first two weeks but a significant rise within two years (32). Only one patient (7%) of our series developed a seizure within two weeks but additional five patients (33%) within the next 4 years (range 1–4 years). Interestingly, all seizures could easily be controlled with a single antiepileptic medication. Awareness of seizures is also important in the long term (31, 33), and some neurosurgeons propose prophylactic antiepileptic medications for 7 days in all patients with PBI (29).

### Glasgow Outcome Score According to LVI and HVI

Several factors were tested for correlation with outcome. Injury type was found to be highly significant. Therefore, differentiating between LVI and HVI seems to be crucial for decision-making and predicting possible outcomes. As expected, the outcome in LVI was significantly better than in HVI. Mortality of 8% with a favourable outcome in 69% was shown in LVI in our study. This is comparable to the reports in the literature where a favourable outcome appears possible in most patients with LVI (14, 15, 27, 34). The concept of differentiation between LVI and HVI was also considered in the narrative review of Wan et al. (29). In contrast to our outcome data, Kumar found significant morbidity in 71% of patients requiring surgery and 43% of patients being left with permanent neurological deficits in their pediatric population (31). The doubled percentage compared with our date is of note, as one would expect, that especially in the pediatric population neurologic deficits have a higher chance to recover. In HVI, mortality was 73% and a favourable outcome could only be achieved in 18%. These results seem worse compared to other studies with Turco showing mortality of 62% and Deng reporting mortality of 55% in firearm-related PBI (26, 35). On the other hand, Deng demonstrated in his series that self-inflicted gunshot wounds to the head accounted for much higher mortality (72%) (26). One has to keep in mind that the rate of self-inflicted injuries was much lower in these studies than in our series. Mortality in firearm injuries (HVI) seems to depend strongly on its intention with self-infliction showing higher mortality than assaults. An explanation for this could be that self-inflicted injuries are usually caused by a point-blank GSW.

### Glasgow Outcome Score According to Clinicoradiological Parameters

Initial GCS and pupillary status are well-known prognostic factors for the outcome in traumatic brain injury (7, 9, 36) and were also confirmed in our study. However, two patients (20%) with mild TBI died, whereas two patients (15%) with severe TBI achieved a favourable outcome. This unusual clinical course was also seen with pupillary status, where five patients (36%) with non-pathological pupillary status had an unfavourable outcome or died. This reflects the importance of giving meticulous caution to patients with PBI.

Penetrating brain injury is in every case a severe injury to the brain but does not obligatory lead to low GCS or pathological pupillary status. Furthermore, bilateral trajectories have been reported as negative prognostic factors in PBI, reflecting the impact of the injury (22, 35). For example, Turco reported in a collective review of GSW to the head overall mortality of 62% and a mortality of 82% in bilateral trajectories. In self-inflicted injuries, bilateral trajectories occurred more frequently as well (35). This is confirmed in our study, where bilateral trajectories accounted for the highest mortality (88%) of all investigated parameters.

Cistern patency on initial CT as a prognostic factor for PBI has been a controversial topic in the literature. Gressot showed no predictive significance for present, compressed, or absent cisterns in GSW to the head (9). Aarabi found the patency of basal cisterns as a significant determinant for the outcome in GSW to the head (7). Muehlschlegel differentiated between present or compressed cisterns as a positive prognostic factor and absent cisterns as a negative prognostic factor (23). However, in our study, ventricle size and cistern patency did not achieve significance.

## Conclusion

Few data exist on solely civilian PBI in western European countries. There are major national geographical differences in PBI worldwide. Furthermore, one has to differentiate between civilian- and combat-related PBI since these patients present with many different and distinguishable variables. Subsequently, in a civilian setting, a favourable outcome is possible in almost half of the patients who succeed in surgery. Differentiating LVI and HVI is of utmost importance in decision-making and predicting outcomes. In patients presenting with bilateral trajectories (penetration crossing the midline), no favourable outcome could be achieved.

### Limitations

This study is associated with different limitations. (1) First, this represents a retrospective study with its known shortcomings. However, one must keep in mind whether a prospective study is feasible in such heterogenous diseases. Furthermore, treatment decisions in these urgent circumstances easily preclude the complete fulfilment of prospective study protocols. (2) Additionally, the number of included patients is small. However, it reflects the frequency of this injury pattern in western European countries. (3) Also, statistical comparisons between small cohorts are fraught and do not allow general and robust conclusions. (4) At last, it is important to note that only patients receiving surgery have been included in this study.

## Data Availability

The raw data supporting the conclusions of this article will be made available by the authors, without undue reservation.
